# Clinical comparison of patients with benign urachal masses versus urachal carcinomas

**DOI:** 10.1186/s40880-016-0173-4

**Published:** 2017-01-07

**Authors:** Xing Bi, Zhiming Wu, Hui Han, Fangjian Zhou

**Affiliations:** 1 Department of Urology, Tumor Hospital affiliated with the Xinjiang Medical University, Urumqi, 830011 Xinjiang P. R. China; 2 Department of Urology, State Key Laboratory of Oncology in South China, Collaborative Innovation Center for Cancer Medicine, Sun Yat-sen University Cancer Center, Guangzhou, 510060 Guangdong P. R. China

**Keywords:** Urachal mass, Urachal carcinoma, Diagnosis

## Abstract

The clinical features of 17 patients with benign urachal masses and 30 patients with urachal carcinoma treated at Sun Yat-sen University Cancer Center were analyzed retrospectively. Univariate analysis indicated that seven parameters differed significantly between the two groups. Binary logistic regression analyses showed that the rate of gross hematuria was significantly higher (*P* = 0.042, Exp[B] = 7.889) and the rate of fatty infiltration of the Retzius space was significantly lower (*P* = 0.006, Exp[B] = 0.028) in patients with urachal carcinoma than in those with benign urachal masses. Gross hematuria and fatty infiltration of the Retzius space may be indications of malignant and benign urachal masses, respectively.

## Background

Urachal masses may be malignant or benign, and the accuracy of preoperative diagnosis is poor [1–3]. In the present study, we retrospectively analyzed the clinical features of patients with both types of disease to identify indicators for accurate preoperative diagnosis to potentially prevent unnecessary resection.

## Patients and methods

Clinical data of patients with urachal masses treated between 2000 and 2015 at Sun Yat-sen University Cancer Center were retrospectively analyzed. Patients were included if they (1) were aged >18 years, (2) had been diagnosed based on pathology, and (3) had lesions located in the dome or midline of the bladder. Additionally, patients with urachal carcinoma were included if (1) there was a sharp demarcation between the tumor and the bladder urothelium and (2) the patient did not have a primary adenocarcinoma of another organ [4].

All patients provided a detailed medical history. The parameters recorded included patient age, sex, the white blood cell count, the percentage of neutrophils, and the presence of gross hematuria, lower urinary tract symptoms (frequency, urgency, and dysuria), a palpable mass, lower abdominal pain (complaints of pain and tenderness), mucinuria, and calcification. Fatty infiltration of the Retzius space was characterized by enhanced patches or strips in the Retzius space, with vague indications of fat surrounding the lesion. Diffuse thickening of the adjacent bladder wall was characterized by thickening of the bladder wall not only around the lesions but also across the whole bladder dome and two side walls, with the peripheral wall gradually thinning to normal. Fatty infiltration and bladder wall thickening were examined using computed tomography (CT). All images were evaluated by two radiologists using the above criteria, with consistent results.

Continuous data were compared using independent-sample *t* test, and categorical data were compared using Chi square test. Factors differing significantly between patients with benign urachal masses and those with urachal carcinoma in the univariate analysis were further subjected to binary logistic regression analysis (likelihood ratio test). A *P* value <0.05 was considered statistically significant.

## Results

The study included 30 patients with urachal carcinoma (the malignant group) and 17 with benign urachal masses (the benign group). The baseline patient characteristics are shown in Table 1. The median age was 45 years (range 22–62 years) in the malignant group and 41 years (range 18–75 years) in the benign group (*P* = 0.362). The malignant group was composed of 21 (70%) men and 9 (30%) women, whereas the benign group was composed of 7 (41%) men and 10 (59%) women.

**Table 1:** Univariate analysis of all 12 parameters compared between patients with benign urachal masses and those with urachal carcinoma

Variable	Benign urachal masses (cases)	Urachal carcinoma (cases)	*P* value
Total	17	30	
Sex			0.053
Male	7	21	
Female	10	9	
Gross hematuria			0.021
Present	6	21	
Absent	11	9	
Lower urinary tract symptoms			0.011
Present	8	4	
Absent	9	26	
Palpable mass			0.632
Present	1	3	
Absent	16	27	
Lower abdominal pain			0.004
Present	8	3	
Absent	9	27	
Mucinuria			0.283
Present	0	2	
Absent	17	28	
White blood cell count			0.001
≥10 × 10^9^/L	7	0	
<10 × 10^9^/L	10	30	
Percentage of neutrophils (%)			0.004
≥70	9	3	
<70	8	27	
Calcification			0.449
Present	6	14	
Absent	11	16	
Fatty infiltration of the Retzius space			<0.001
Present	12	1	
Absent	5	29	
Diffuse thickening of adjacent bladder wall			<0.001
Present	9	0	
Absent	8	30	

**Fig. 1:**
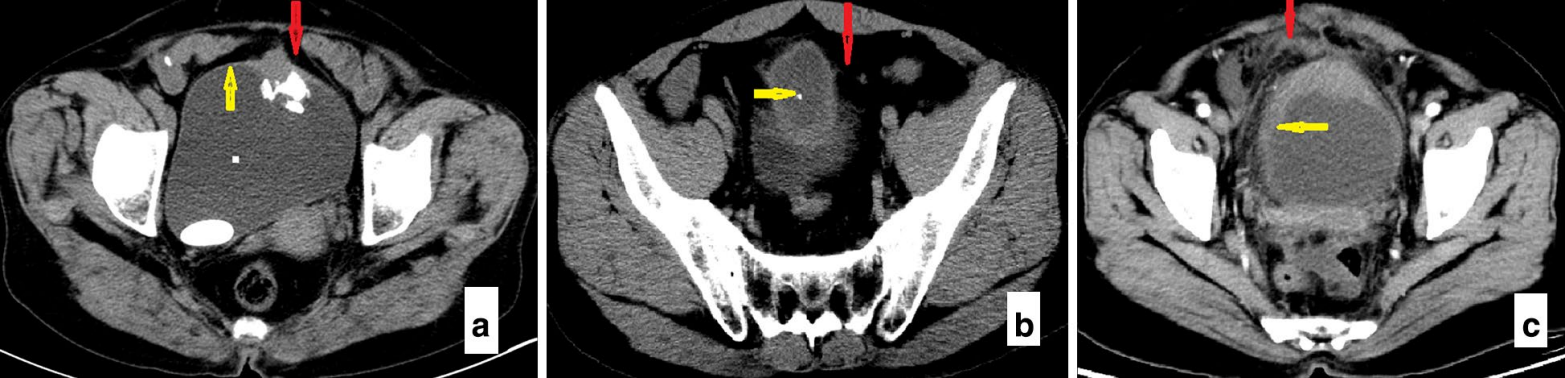
Computed tomography (CT) manifestations of a urachal carcinoma and a benign urachal mass. **a** On an axial view of a urachal carcinoma, the *red arrow* indicates the Retzius space without fat infiltration, and the *yellow arrow* indicates a lack of thickening of the surrounding bladder wall. **b** On an axial view of a urachal carcinoma, the *red arrow* indicates the Retzius space without fat infiltration, and the *yellow arrow* indicates calcification in central lesions. **c** On an axial view of a benign urachal mass, the *red arrow* indicates fatty infiltration of the Retzius space, characterized by enhancements of some patches or strips and vague indications of fat surrounding the lesions, and the *yellow arrow* indicates diffuse thickening of the bladder wall not only around the lesions but also across the whole bladder dome and two side walls, with the peripheral wall gradually thinning to normal

Of the 30 patients with urachal carcinoma, 29 had adenocarcinomas, and 1 had a sarcomatoid carcinoma combined with adenocarcinoma. Of the 17 patients with benign urachal masses, 15 had infected urachal cysts with chronic inflammation, and 2 had xanthogranuloma. Imaging indices are presented in Fig. 1.

Table 1 shows the univariate analysis of all 12 parameters compared between the malignant and benign groups. No significant differences were observed in age, sex, the presence of a palpable mass, mucinuria, or calcification. The rate of gross hematuria was higher in the malignant group than in the benign group (*P* = 0.021). In contrast, the rates of lower urinary tract symptoms (*P* = 0.011), lower abdominal pain (*P* = 0.004), fatty infiltration of the Retzius space (*P* < 0.001), and diffuse thickening of the adjacent bladder wall (*P* < 0.001) as well as the percentage of neutrophils (*P* = 0.004) and the white blood cell count (*P* = 0.001) were significantly higher in the benign group than in the malignant group.

Binary logistic regression analysis of the seven parameters that differed significantly between the two groups in the univariate analysis showed that the rate of gross hematuria was significantly higher (*P* = 0.042, Exp[B] = 7.889) and the rate of fatty infiltration of the Retzius space was significantly lower (*P* = 0.006, Exp[B] = 0.028) in the malignant group than in the benign group.

## Discussion

Urachal carcinoma is a rare but highly malignant epithelial cancer that is difficult to distinguish from a benign urachal mass [5]. Urachal inflammation and certain benign urachal neoplasms, including adenomas, fibromas, villous adenomas, and mucinous cystadenomas, can form masses.

Urachal carcinoma has been reported more frequently in men than in women (1.49:1), and most patients are older than 50 years [6, 7]. However, in the present study, the median age of the patients with urachal carcinoma was 45 years, with no significant differences between the two groups in terms of age or sex.

Multivariate analysis identified two parameters that differed significantly between the benign and the malignant groups. Gross hematuria was significantly associated with urachal carcinoma (*P* = 0.042), whereas fatty infiltration of the Retzius space was significantly associated with benign urachal masses (*P* = 0.006). These results were consistent with those of previous reports, in which hematuria was a common risk factor for malignancy [8, 9]. Fatty infiltration of the Retzius space has also been reported to be an important indirect sign of inflammation in patients with urachal cysts [10].

This study had several limitations, including its retrospective nature and the small number of patients. In addition, all of the benign urachal masses were inflamed. Thus, these results may not apply to all types of benign neoplasms.

## Conclusions

This study showed that gross hematuria was significantly associated with urachal carcinoma, whereas fatty infiltration of the Retzius space was significantly associated with benign urachal masses. Additional studies are needed to verify these findings.
